# Supplementary material for: Emergence and Persistent Circulation of Highly Pathogenic Avian Influenza Virus A (H5N8) in Kosovo, May 2021–May 2022
Source: Microorganisms. 2023 Sep 2;11(9):2226. doi: 10.3390/microorganisms11092226 (PMC10534373; doi:10.3390/microorganisms11092226)
Supplement: Supplementary file 1 [file microorganisms-11-02226-s001.zip › Supplementary Table S1. Primer_probe sequences used in the initial screening of the suspected outbreaks in the Kosovo Food and Veterinary Laboratory.pdf]

Table S1. Primer/probe sequences used in the initial screening of the suspected outbreaks in the Kosovo Food and Veterinary Laboratory

Assay	Oligo name	Sequence (5′ to 3′)	Final conc.(μM) in 25μl of final volume
AI type A (Heine, et al., 2015)	Forw IVA D161M	AGA TGA GYC TTC TAA CCG AGG TCG	0.9
	Rev IVA D162M1	TGC AAA AAC ATC YTC AAG TCT CTG	0.225
	Rev IVA D162M2	TGC AAA CAC ATC YTC AAG TCT CTG	0.225
	Rev IVA D162M3	TGC AAA GAC ATC YTC AAG TCT CTG	0.225
	Rev IVA D162M4	TGC AAA TAC ATC YTC AAG TCT CTG	0.225
	Probe IVA MA	FAM-TCA GGC CCC CTC AAA GCC GA-TAMRA	0.25
Eurasian H5 (Slomka et al., 2007)	Forw H5LH1	ACA TAT GAC TAC CCA CAR TAT TCA G	0.4
	Rev H5RH1	AGA CCA GCT AYC ATG ATT GC	0.4
	Probe H5PRO	FAM- TCW ACA GTG GCG AGT TCC CTA GCA-TAMRA	0.3
Eurasian H7 (Van Borm et al., 2010 modified)	Forw FeurH7	GYA GYG GYT ACA AAG ATG TG	0.9
	Rev ReurH7	GAA GAC AAG GCC CAT TGC AA	0.9
	Probe IAV-HA7-CODA	FAM-TGG TTT AGC TTC GGG GCA TCA TG-BHQ1	0.2
N1 (Payungporn, et al., 2006)	For N1F2	GTT TGA GTC TGT TGC TTG GTC	0.4
	Rev N1R1	TGA TAG TGT CTG TTA TTA TGC C	0.4
	Probe N1	VIC-TTG TAT TTC AAT ACA GCC AC-(MGB)	0.2
N8 (James, et al., 2019)	For N8	YCC CTG YTT TTG GGT CGA AAT GAT	0.8
	Rev N8	GCT CCA TCG TGC CAT GAC CA	0.8
	Probe N8	FAM – TCT AGT AGC TCC ATT GTA ATG TGT GGA GT – BHQ1	0.4
