# Supplementary figures and images for: Emergence and Persistent Circulation of Highly Pathogenic Avian Influenza Virus A (H5N8) in Kosovo, May 2021–May 2022
Source: Microorganisms. 2023 Sep 2;11(9):2226. doi: 10.3390/microorganisms11092226 (PMC10534373; doi:10.3390/microorganisms11092226)

PB2 gene

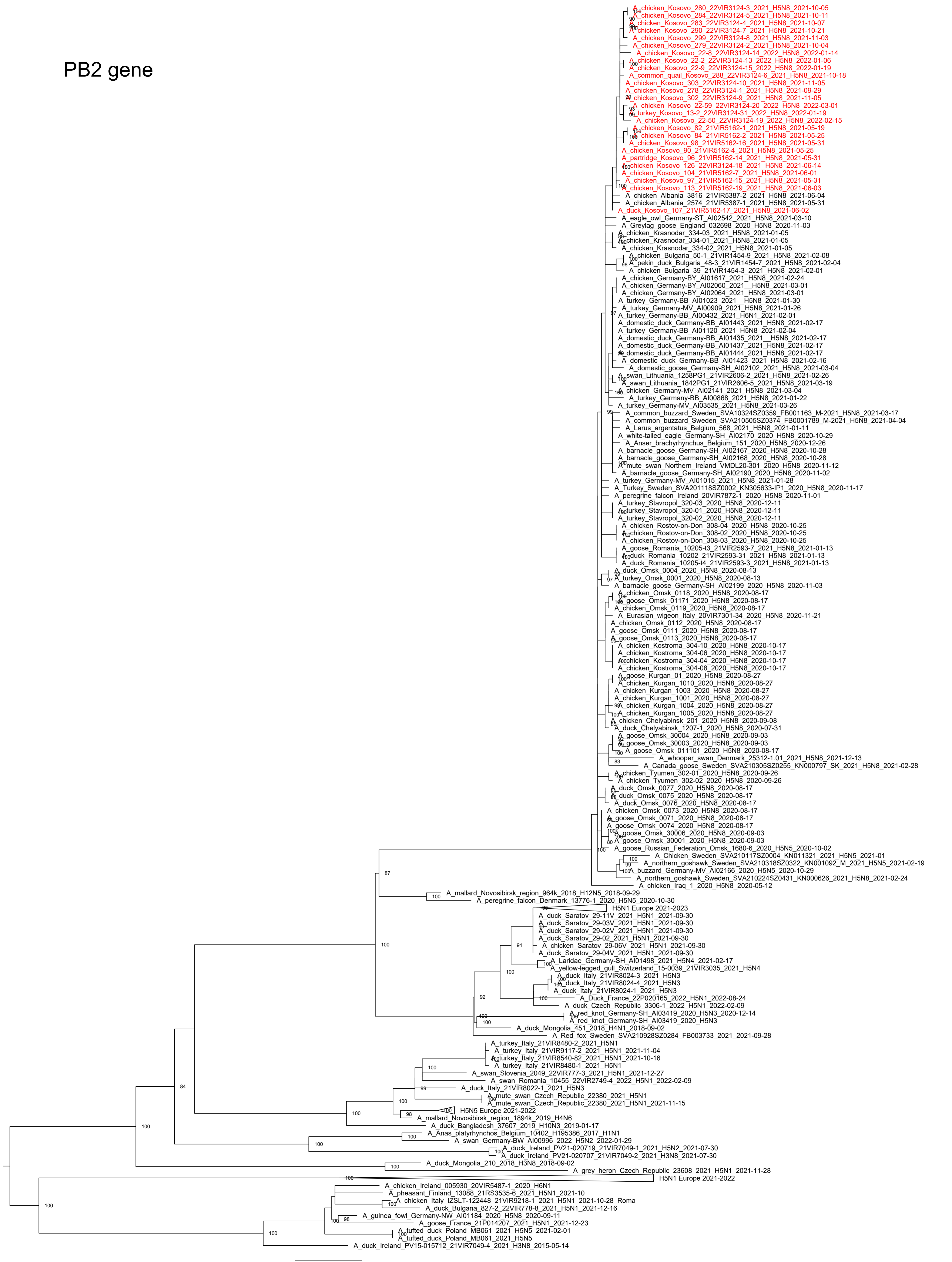

Supplement: Supplementary file 1 [file microorganisms-11-02226-s001.zip › Supplementary Figure S1_PB2_tree.pdf]

# PB1 gene

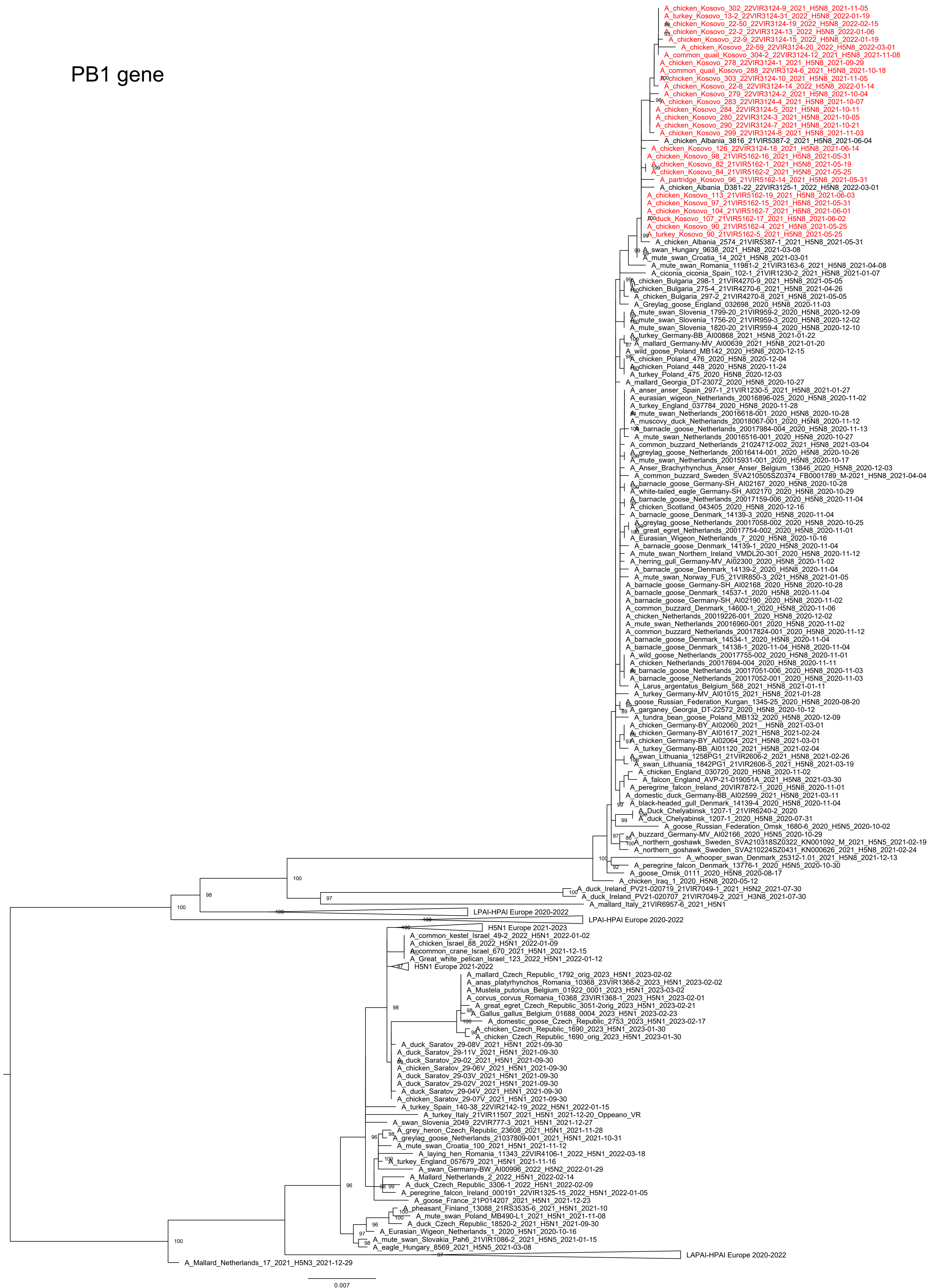

Supplement: Supplementary file 1 [file microorganisms-11-02226-s001.zip › Supplementary Figure S2_PB1_tree.pdf]

PA gene

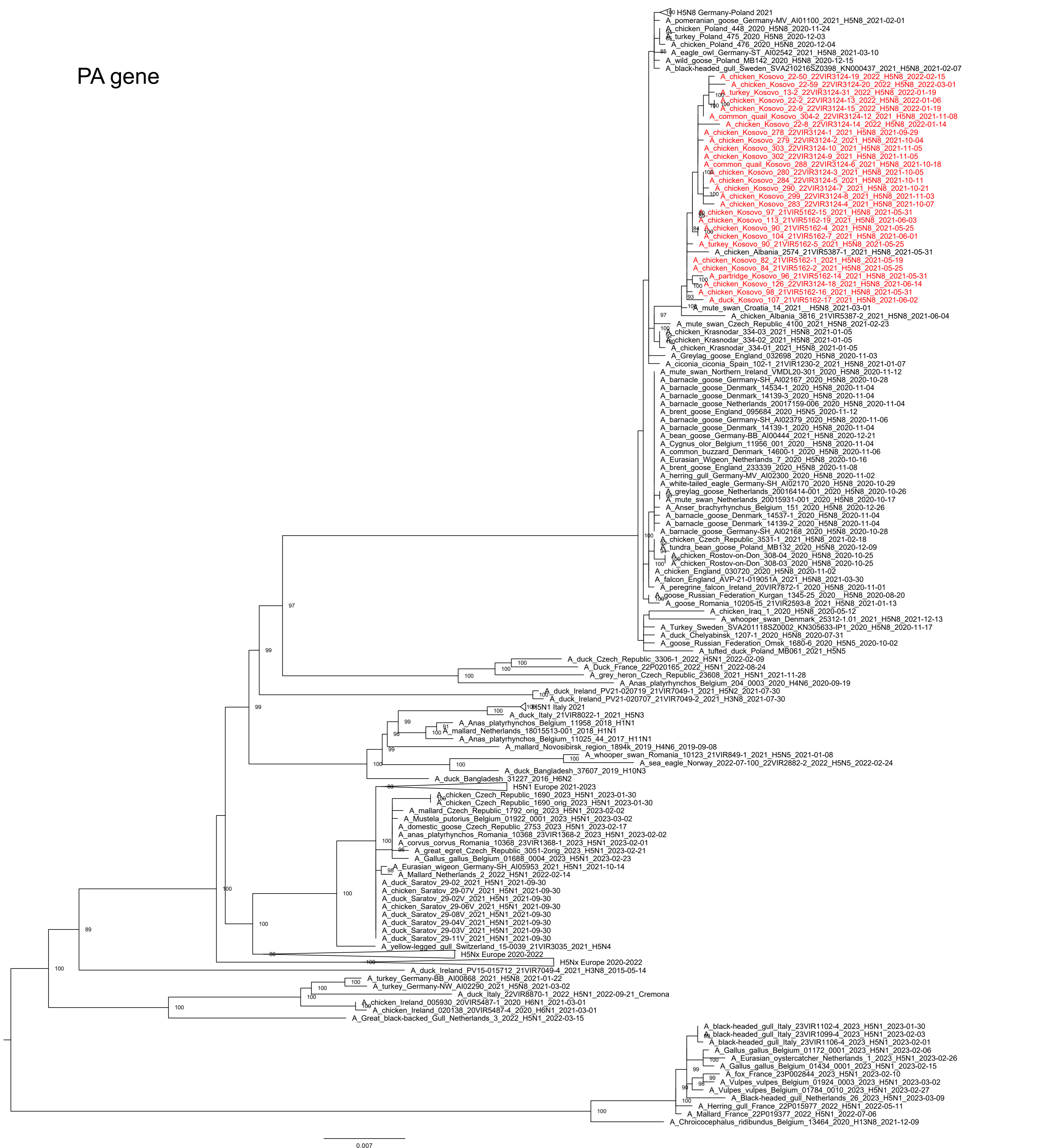

Supplement: Supplementary file 1 [file microorganisms-11-02226-s001.zip › Supplementary Figure S3_PA_tree.pdf]

NA gene - N8 subtype

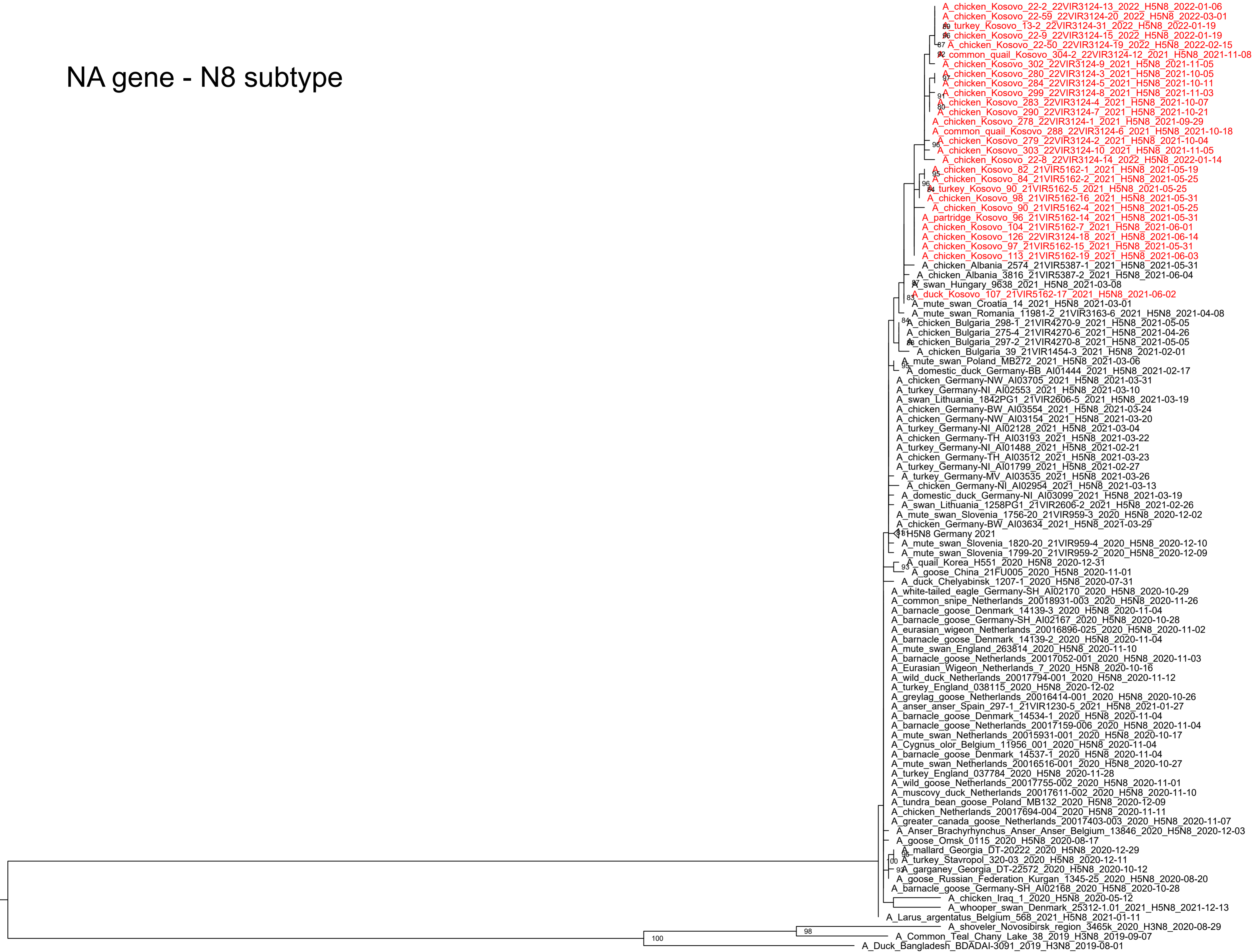

Supplement: Supplementary file 1 [file microorganisms-11-02226-s001.zip › Supplementary Figure S5_NA-N8_tree.pdf]

M gene

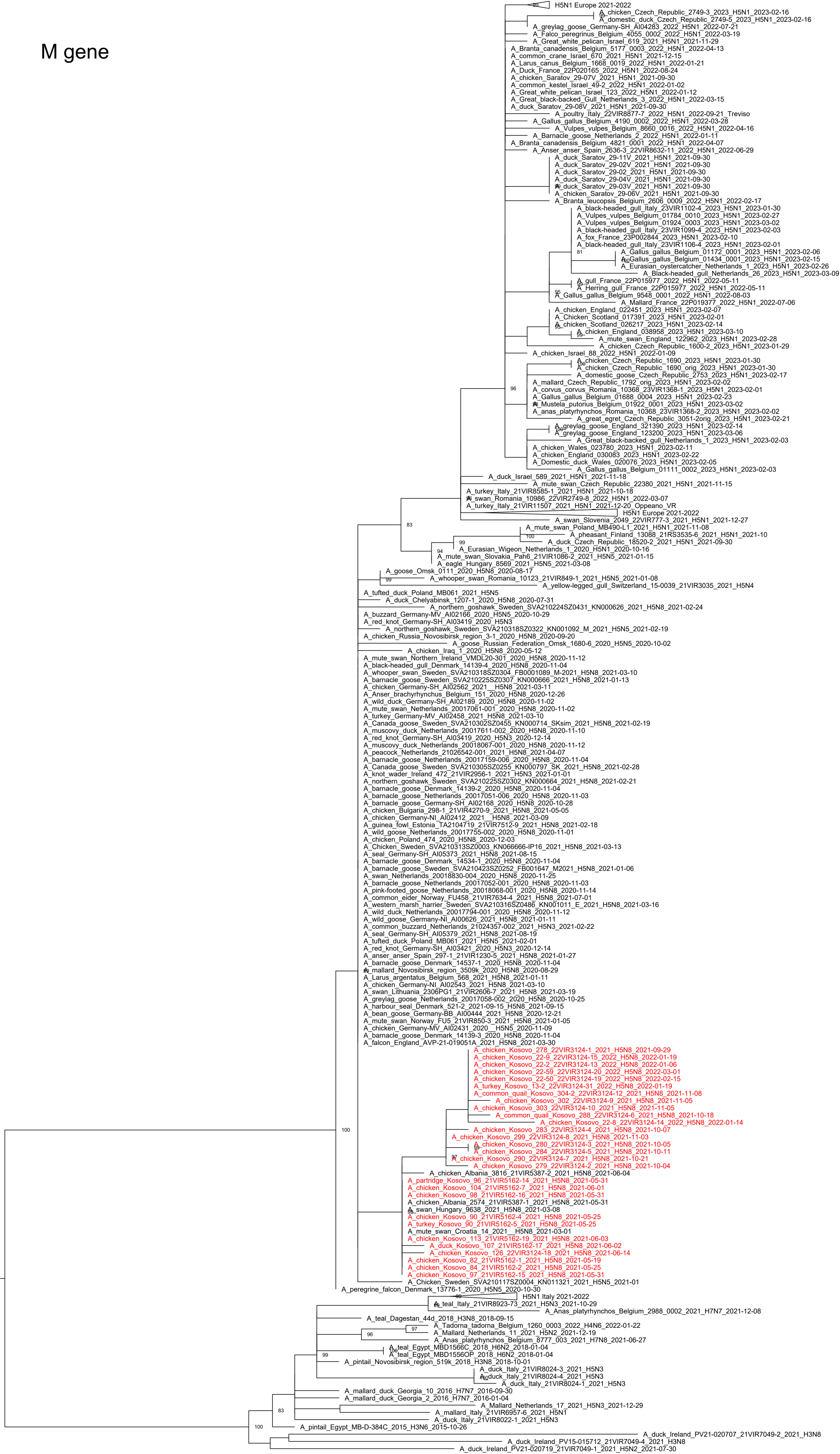

Supplement: Supplementary file 1 [file microorganisms-11-02226-s001.zip › Supplementary Figure S6_M_tree.pdf]

NS gene

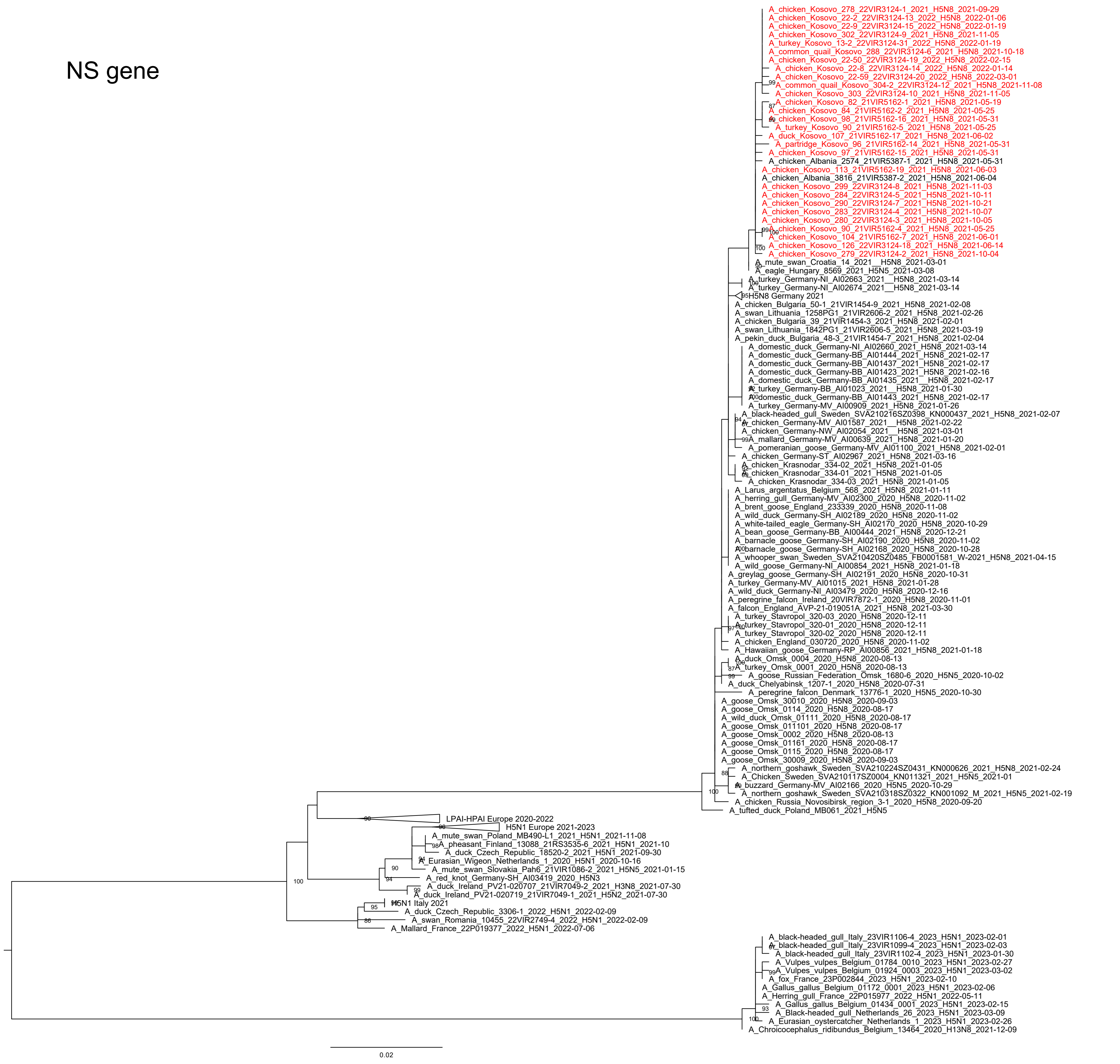

Supplement: Supplementary file 1 [file microorganisms-11-02226-s001.zip › Supplementary Figure S7_NS_tree.pdf]
