# Supplementary material for: Emergence and Persistent Circulation of Highly Pathogenic Avian Influenza Virus A (H5N8) in Kosovo, May 2021–May 2022
Source: Microorganisms. 2023 Sep 2;11(9):2226. doi: 10.3390/microorganisms11092226 (PMC10534373; doi:10.3390/microorganisms11092226)
Supplement: Supplementary file 1 [file Supplementary Figure S4_NP_tree.pdf › Supplementary Figure S4_NP_tree.pdf]

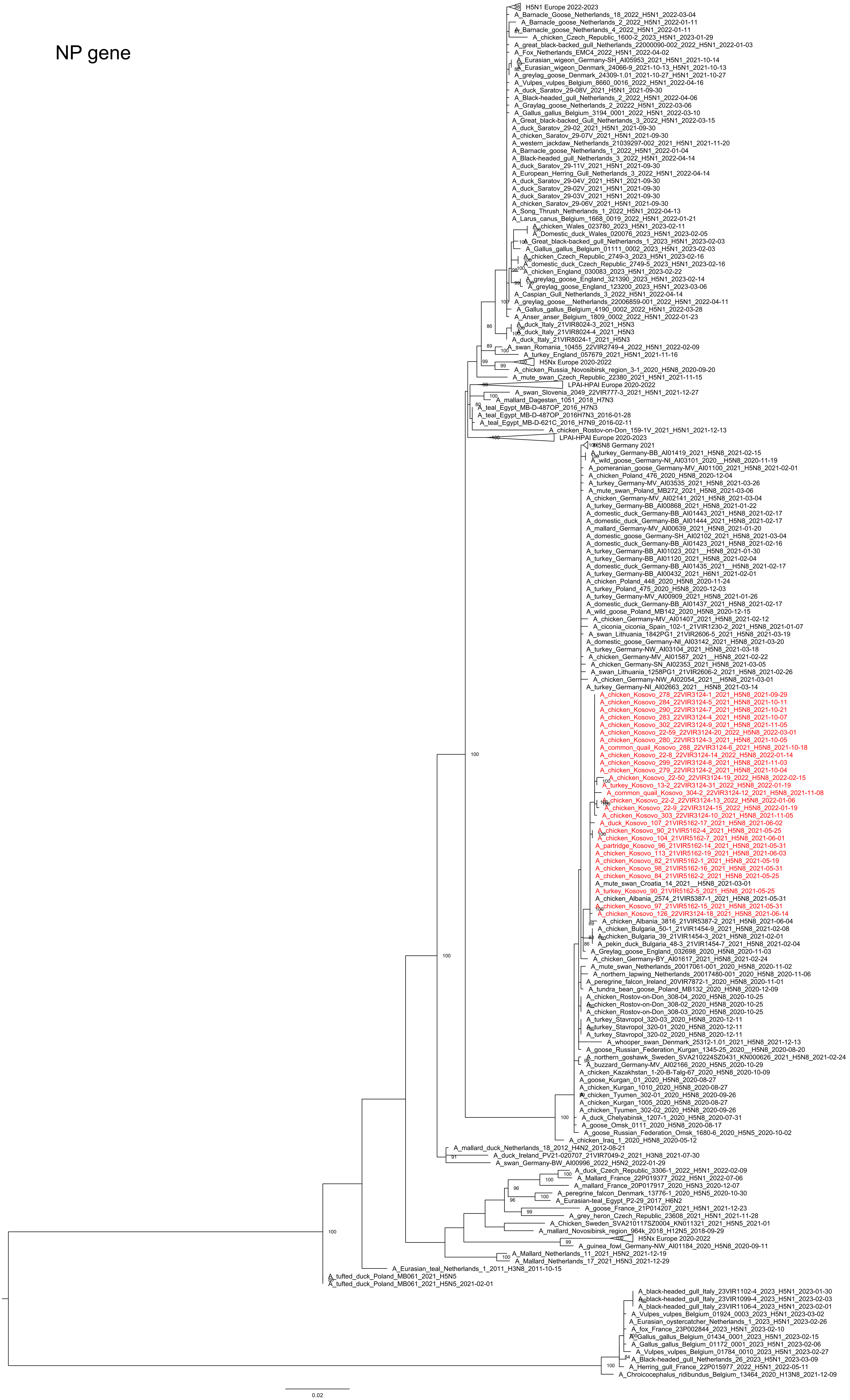
NP gene
H5N1 Europe 2022-2023
A_Barnacle_Goose_Netherlands_18_2022_H5N1_2022-03-04
A_Barnacle_goose_Netherlands_2_2022_H5N1_2022-01-11
A_Barnacle_goose_Netherlands_4_2022_H5N1_2022-01-11
A_chicken_Czech_Republic_1600-2_2023_H5N1_2023-01-29
A_great_black-backed_gull_Netherlands_22000090-002_2022_H5N1_2022-01-03
A_Fox_Netherlands_EMC4_2022_H5N1_2022-04-02
A_Eurasian_wigeon_Germany-SH_AI05953_2021_H5N1_2021-10-14
A_Eurasian_wigeon_Denmark_24066-9_2021-10-13_H5N1_2021-10-13
A_greylag_goose_Denmark_24309-1.01_2021-10-27_H5N1_2021-10-27
A_Vulpes_vulpes_Belgium_8660_0016_2022_H5N1_2022-04-16
A_duck_Saratov_29-08V_2021_H5N1_2021-09-30
A_Black-headed_gull_Netherlands_2_2022_H5N1_2022-04-06
A_Graylag_goose_Netherlands_2_20222_H5N1_2022-03-06
A_Gallus_gallus_Belgium_3194_0001_2022_H5N1_2022-03-10
A_Great_black-backed_Gull_Netherlands_3_2022_H5N1_2022-03-15
A_duck_Saratov_29-02_2021_H5N1_2021-09-30
A_chicken_Saratov_29-07V_2021_H5N1_2021-09-30
A_western_jackdaw_Netherlands_21039297-002_2021_H5N1_2021-11-20
A_Barnacle_goose_Netherlands_1_2022_H5N1_2022-01-04
A_Black-headed_gull_Netherlands_3_2022_H5N1_2022-04-14
A_duck_Saratov_29-11V_2021_H5N1_2021-09-30
A_European_Herring_Gull_Netherlands_3_2022_H5N1_2022-04-14
A_duck_Saratov_29-04V_2021_H5N1_2021-09-30
A_duck_Saratov_29-02V_2021_H5N1_2021-09-30
A_duck_Saratov_29-03V_2021_H5N1_2021-09-30
A_chicken_Saratov_29-06V_2021_H5N1_2021-09-30
A_Song_Thrush_Netherlands_1_2022_H5N1_2022-04-13
A_Larus_canus_Belgium_1668_0019_2022_H5N1_2022-01-21
A_chicken_Wales_023780_2023_H5N1_2023-02-11
A_Domestic_duck_Wales_020076_2023_H5N1_2023-02-05
A_Great_black-backed_gull_Netherlands_1_2023_H5N1_2023-02-03
A_Gallus_gallus_Belgium_01111_0002_2023_H5N1_2023-02-03
A_chicken_Czech_Republic_2749-3_2023_H5N1_2023-02-16
A_domestic_duck_Czech_Republic_2749-5_2023_H5N1_2023-02-16
A_chicken_England_030083_2023_H5N1_2023-02-22
A_greylag_goose_England_321390_2023_H5N1_2023-02-14
A_greylag_goose_England_123200_2023_H5N1_2023-03-06
A_Caspian_Gull_Netherlands_3_2022_H5N1_2022-04-14
A_greylag_goose__Netherlands_22006859-001_2022_H5N1_2022-04-11
A_Gallus_gallus_Belgium_4190_0002_2022_H5N1_2022-03-28
A_Anser_anser_Belgium_1809_0002_2022_H5N1_2022-01-23
A_duck_Italy_21VIR8024-3_2021_H5N3
A_duck_Italy_21VIR8024-4_2021_H5N3
A_duck_Italy_21VIR8024-1_2021_H5N3
A_swan_Romania_10455_22VIR2749-4_2022_H5N1_2022-02-09
A_turkey_England_057679_2021_H5N1_2021-11-16
H5Nx Europe 2020-2022
A_chicken_Russia_Novosibirsk_region_3-1_2020_H5N8_2020-09-20
A_mute_swan_Czech_Republic_22380_2021_H5N1_2021-11-15
LPAI-HPAI Europe 2020-2022
A_swan_Slovenia_2049_22VIR777-3_2021_H5N1_2021-12-27
A_mallard_Dagestan_1051_2018_H7N3
A_teal_Egypt_MB-D-487OP_2016_H7N3
A_teal_Egypt_MB-D-487OP_2016H7N3_2016-01-28
A_teal_Egypt_MB-D-621C_2016_H7N9_2016-02-11
A_chicken_Rostov-on-Don_159-1V_2021_H5N1_2021-12-13
LPAI-HPAI Europe 2020-2023
H5N8 Germany 2021
A_turkey_Germany-BB_AI01419_2021_H5N8_2021-02-15
A_wild_goose_Germany-NI_AI03101_2020__H5N8_2020-11-19
A_pomeranian_goose_Germany-MV_AI01100_2021_H5N8_2021-02-01
A_chicken_Poland_476_2020_H5N8_2020-12-04
A_turkey_Germany-MV_AI03535_2021_H5N8_2021-03-26
A_mute_swan_Poland_MB272_2021_H5N8_2021-03-06
A_chicken_Germany-MV_AI02141_2021_H5N8_2021-03-04
A_turkey_Germany-BB_AI00868_2021_H5N8_2021-01-22
A_domestic_duck_Germany-BB_AI01443_2021_H5N8_2021-02-17
A_domestic_duck_Germany-BB_AI01444_2021_H5N8_2021-02-17
A_mallard_Germany-MV_AI00639_2021_H5N8_2021-01-20
A_domestic_goose_Germany-SH_AI02102_2021_H5N8_2021-03-04
A_domestic_duck_Germany-BB_AI01423_2021_H5N8_2021-02-16
A_turkey_Germany-BB_AI01023_2021__H5N8_2021-01-30
A_turkey_Germany-BB_AI01120_2021_H5N8_2021-02-04
A_domestic_duck_Germany-BB_AI01435_2021__H5N8_2021-02-17
A_turkey_Germany-BB_AI00432_2021_H6N1_2021-02-01
A_chicken_Poland_448_2020_H5N8_2020-11-24
A_turkey_Poland_475_2020_H5N8_2020-12-03
A_turkey_Germany-MV_AI00909_2021_H5N8_2021-01-26
A_domestic_duck_Germany-BB_AI01437_2021_H5N8_2021-02-17
A_wild_goose_Poland_MB142_2020_H5N8_2020-12-15
A_chicken_Germany-MV_AI01407_2021_H5N8_2021-02-12
A_ciconia_ciconia_Spain_102-1_21VIR1230-2_2021_H5N8_2021-01-07
A_swan_Lithuania_1842PG1_21VIR2606-5_2021_H5N8_2021-03-19
A_domestic_goose_Germany-NI_AI03142_2021_H5N8_2021-03-20
A_turkey_Germany-NW_AI03104_2021_H5N8_2021-03-18
A_chicken_Germany-MV_AI01587_2021__H5N8_2021-02-22
A_chicken_Germany-SN_AI02353_2021_H5N8_2021-03-05
A_swan_Lithuania_1258PG1_21VIR2606-2_2021_H5N8_2021-02-26
A_chicken_Germany-NW_AI02054_2021__H5N8_2021-03-01
A_turkey_Germany-NI_AI02663_2021__H5N8_2021-03-14
A_chicken_Kosovo_278_22VIR3124-1_2021_H5N8_2021-09-29
A_chicken_Kosovo_284_22VIR3124-5_2021_H5N8_2021-10-11
A_chicken_Kosovo_290_22VIR3124-7_2021_H5N8_2021-10-21
A_chicken_Kosovo_283_22VIR3124-4_2021_H5N8_2021-10-07
A_chicken_Kosovo_302_22VIR3124-9_2021_H5N8_2021-11-05
A_chicken_Kosovo_22-59_22VIR3124-20_2022_H5N8_2022-03-01
A_chicken_Kosovo_280_22VIR3124-3_2021_H5N8_2021-10-05
A_common_quail_Kosovo_288_22VIR3124-6_2021_H5N8_2021-10-18
A_chicken_Kosovo_22-8_22VIR3124-14_2022_H5N8_2022-01-14
A_chicken_Kosovo_299_22VIR3124-8_2021_H5N8_2021-11-03
A_chicken_Kosovo_279_22VIR3124-2_2021_H5N8_2021-10-04
A_chicken_Kosovo_22-50_22VIR3124-19_2022_H5N8_2022-02-15
A_turkey_Kosovo_13-2_22VIR3124-31_2022_H5N8_2022-01-19
A_common_quail_Kosovo_304-2_22VIR3124-12_2021_H5N8_2021-11-08
A_chicken_Kosovo_22-2_22VIR3124-13_2022_H5N8_2022-01-06
A_chicken_Kosovo_22-9_22VIR3124-15_2022_H5N8_2022-01-19
A_chicken_Kosovo_303_22VIR3124-10_2021_H5N8_2021-11-05
A_duck_Kosovo_107_21VIR5162-17_2021_H5N8_2021-06-02
A_chicken_Kosovo_90_21VIR5162-4_2021_H5N8_2021-05-25
A_chicken_Kosovo_104_21VIR5162-7_2021_H5N8_2021-06-01
A_partridge_Kosovo_96_21VIR5162-14_2021_H5N8_2021-05-31
A_chicken_Kosovo_113_21VIR5162-19_2021_H5N8_2021-06-03
A_chicken_Kosovo_82_21VIR5162-1_2021_H5N8_2021-05-19
A_chicken_Kosovo_98_21VIR5162-16_2021_H5N8_2021-05-31
A_chicken_Kosovo_84_21VIR5162-2_2021_H5N8_2021-05-25
A_mute_swan_Croatia_14_2021__H5N8_2021-03-01
A_turkey_Kosovo_90_21VIR5162-5_2021_H5N8_2021-05-25
A_chicken_Albania_2574_21VIR5387-1_2021_H5N8_2021-05-31
A_chicken_Kosovo_97_21VIR5162-15_2021_H5N8_2021-05-31
A_chicken_Kosovo_126_22VIR3124-18_2021_H5N8_2021-06-14
A_chicken_Albania_3816_21VIR5387-2_2021_H5N8_2021-06-04
A_chicken_Bulgaria_50-1_21VIR1454-9_2021_H5N8_2021-02-08
A_chicken_Bulgaria_39_21VIR1454-3_2021_H5N8_2021-02-01
A_pekin_duck_Bulgaria_48-3_21VIR1454-7_2021_H5N8_2021-02-04
A_Greylag_goose_England_032698_2020_H5N8_2020-11-03
A_chicken_Germany-BY_AI01617_2021_H5N8_2021-02-24
A_mute_swan_Netherlands_20017061-001_2020_H5N8_2020-11-02
A_northern_lapwing_Netherlands_20017480-001_2020_H5N8_2020-11-06
A_peregrine_falcon_Ireland_20VIR7872-1_2020_H5N8_2020-11-01
A_tundra_bean_goose_Poland_MB132_2020_H5N8_2020-12-09
A_chicken_Rostov-on-Don_308-04_2020_H5N8_2020-10-25
A_chicken_Rostov-on-Don_308-02_2020_H5N8_2020-10-25
A_chicken_Rostov-on-Don_308-03_2020_H5N8_2020-10-25
A_turkey_Stavropol_320-03_2020_H5N8_2020-12-11
A_turkey_Stavropol_320-01_2020_H5N8_2020-12-11
A_turkey_Stavropol_320-02_2020_H5N8_2020-12-11
A_whooper_swan_Denmark_25312-1.01_2021_H5N8_2021-12-13
A_goose_Russian_Federation_Kurgan_1345-25_2020__H5N8_2020-08-20
A_northern_goshawk_Sweden_SVA210224SZ0431_KN000626_2021_H5N8_2021-02-24
A_buzzard_Germany-MV_AI02166_2020_H5N5_2020-10-29
A_chicken_Kazakhstan_1-20-B-Talg-67_2020_H5N8_2020-10-09
A_goose_Kurgan_01_2020_H5N8_2020-08-27
A_chicken_Kurgan_1010_2020_H5N8_2020-08-27
A_chicken_Tyumen_302-01_2020_H5N8_2020-09-26
A_chicken_Kurgan_1005_2020_H5N8_2020-08-27
A_chicken_Tyumen_302-02_2020_H5N8_2020-09-26
A_duck_Chelyabinsk_1207-1_2020_H5N8_2020-07-31
A_goose_Omsk_0111_2020_H5N8_2020-08-17
A_goose_Russian_Federation_Omsk_1680-6_2020_H5N5_2020-10-02
A_chicken_Iraq_1_2020_H5N8_2020-05-12
A_mallard_duck_Netherlands_18_2012_H4N2_2012-08-21
A_duck_Ireland_PV21-020707_21VIR7049-2_2021_H3N8_2021-07-30
A_swan_Germany-BW_AI00996_2022_H5N2_2022-01-29
A_duck_Czech_Republic_3306-1_2022_H5N1_2022-02-09
A_Mallard_France_22P019377_2022_H5N1_2022-07-06
A_mallard_France_20P017917_2020_H5N3_2020-12-07
A_peregrine_falcon_Denmark_13776-1_2020_H5N5_2020-10-30
A_Eurasian-teal_Egypt_P2-29_2017_H6N2
A_goose_France_21P014207_2021_H5N1_2021-12-23
A_grey_heron_Czech_Republic_23608_2021_H5N1_2021-11-28
A_Chicken_Sweden_SVA210117SZ0004_KN001321_2021_H5N5_2021-01
A_mallard_Novosibirsk_region_964k_2018_H12N5_2018-09-29
H5Nx Europe 2020-2022
A_guinea_fowl_Germany-NW_AI01184_2020_H5N8_2020-09-11
A_Mallard_Netherlands_11_2021_H5N2_2021-12-19
A_Mallard_Netherlands_17_2021_H5N3_2021-12-29
A_Eurasian_teal_Netherlands_1_2011_H3N8_2011-10-15
A_tufted_duck_Poland_MB061_2021_H5N5
A_tufted_duck_Poland_MB061_2021_H5N5_2021-02-01
A_black-headed_gull_Italy_23VIR1102-4_2023_H5N1_2023-01-30
A_black-headed_gull_Italy_23VIR1099-4_2023_H5N1_2023-02-03
A_black-headed_gull_Italy_23VIR1106-4_2023_H5N1_2023-02-01
A_Vulpes_vulpes_Belgium_01924_0003_2023_H5N1_2023-03-02
A_Eurasian_oystercatcher_Netherlands_1_2023_H5N1_2023-02-26
A_fox_France_23P002844_2023_H5N1_2023-02-10
A_Gallus_gallus_Belgium_01434_0001_2023_H5N1_2023-02-15
A_Gallus_gallus_Belgium_01172_0001_2023_H5N1_2023-02-06
A_Vulpes_vulpes_Belgium_01784_0010_2023_H5N1_2023-02-27
A_Black-headed_gull_Netherlands_26_2023_H5N1_2023-03-09
A_Herring_gull_France_22P015977_2022_H5N1_2022-05-11
A_Chroicocephalus_ridibundus_Belgium_13464_2020_H13N8_2021-12-09
0.02
